# The Effects of Intrusion of Anterior Teeth by Skeletal Anchorage in Deep Bite Patients; A Systematic Review and Meta-Analysis

**DOI:** 10.3390/biomimetics8010101

**Published:** 2023-03-02

**Authors:** Erfan Bardideh, Golnaz Tamizi, Hooman Shafaee, Abdolrasoul Rangrazi, Mahsa Ghorbani, Navid Kerayechian

**Affiliations:** 1 Dental Research Center, Mashhad University of Medical Sciences, Mashhad 9177948959, Iran; 2 School of Dentistry, Mashhad University of Medical Sciences, Mashhad 9177948959, Iran; 3 Dental Materials Research Center, Mashhad University of Medical Sciences, Mashhad 9177948959, Iran; 4 Department of Orthodontics, School of Dentistry, Mashhad University of Medical Sciences, Mashhad 9177948959, Iran; 5 Department of Biomaterials and Biomimetics, New York University, New York, NY 10011, USA

**Keywords:** intrusion, anterior teeth, skeletal anchorage, deep bite, Burstone’s intrusion arch, utility arch, mini-screw

## Abstract

Background: Deep bite is known as one of the most common malocclusions, and its treatment and retention are often challenging. The use of mini-screws has been suggested as an ideal method for the intrusion of incisors in deep-bite patients. Still, there are conflicting reports regarding the superiority of this method compared to other common treatments. Aim: The aim of this systematic review and meta-analysis was to evaluate the effects of the intrusion of anterior teeth by skeletal anchorage in deep bite patients. Methods: From the beginning to 15 September 2022, articles on the topic of interest were searched in electronic databases including PubMed, Web of Science, Scopus, EMBASE, and Cochrane’s CENTRAL. Additionally, a hand search for pertinent studies and a search of the grey literature were carried out. After the selection of eligible studies, data extraction was performed using piloted forms. Inverse-variance random-effects meta-analyses were used to combine the outcome measures of dental indices, skeletal cephalometric indices, and dental cephalometric indices. Results: A total of 15 studies (6 RCT; 9 CCT) were included in the systematic review and 14 were used in the meta-analyses. The differences in overbite changes (MD = −0.45, *p* = 0.04), true incisor intrusion [u1-pp] (MD = −0.62, *p* = 0.003) and molar extrusion [u6-pp] (MD = −0.40, *p* = 0.01) were statistically significant and TADs showed better treatment results than other intrusion methods (segmented intrusion arch, utility arch, J hook headgear). No significant differences regarding overjet, molar and incisor tipping, and skeletal indices between mini-screw and other intrusion methods could be found. Conclusion: The use of mini-screws leads to lower overbite and higher true intrusion (about 0.45 and 0.62 mm, respectively) compared to the use of other methods for intruding upper incisors. Furthermore, the effect of TAD on extrusion of molar teeth is less (by 0.4 mm) than other methods.

## 1. Introduction

Deep bite refers to increasing the vertical overlap between the upper and lower incisors. The ideal overbite range in a normal occlusion is between 2 and 4 mm, or better said, between 5% and 25% of the overall crown height of the lower incisor [1]. Even a range from 25% to 40% can be considered normal if it is not associated with functional problems during various movements of the temporomandibular joint (TMJ). However, an overlap of more than 40% of the lower incisors’ crown height should be considered a deep bite because it has the potential to have detrimental effects on the overall dental health of adjacent periodontal structures and the TMJ [2]. Deep bite is known as one of the most common malocclusions, and its treatment and retention are often challenging. According to the National Health and Nutritional Estimates survey III, the prevalence of deep overbite is estimated to be in 49% of the general population [3].

The etiology of deep bite is multifactorial, which includes several hidden skeletal or dental disorders. Based on this, deep bite is not a disease but a clinical manifestation of existing skeletal or dental disorders. From an evolutionary point of view, skeletal or dental overbite is caused by genetic factors, environmental factors, or a combination of both [4].

The difference in the growth of the jaw bone in the sagittal and vertical dimensions can cause deep overbite; skeletal deep bite is often seen in a horizontal growth pattern and its features include 1. maxilla and mandible bone growth disorder, 2. convergent rotation of jaw bases, or 3. the height of the ramus of the mandible is low. In this situation, the front height of the face, especially the lower third of the face, is often short. On the other hand, dental deep bite shows the supraocclusion of incisors, infraocclusion of molars, or a combination of the two [5,6].

Other factors that may affect these conditions include changes in the morphology of the teeth, early loss of permanent teeth that causes lingual collapse of the anterior teeth of the maxilla or mandible, the mesiodistal width of the anterior teeth, and the natural deepening of the bite due to aging [7,8].

Deep bite could introduce several functional and aesthetical problems, including problems with TMJ, dentition, periodontium, aesthetics, function, incisal attrition, anterior gingival loss, clenching, headache, and tinnitus. Deep bite treatment is a biomimetic orthodontic treatment that counteracts the overgrowth of incisors and/or undergrowth of molars by mimicking the growth pattern of an individual with normal growth pattern. It could also alleviate the problems associated with this malocclusion and could help the patient achieve better soft tissue and hard tissue harmony [9,10,11].

There are different treatments for deep bite correction, which differ depending on the etiology and severity of the problem, the amount of remaining growth, the relationship of the teeth with the adjacent soft tissue structures, the vertical growth pattern, and cosmetic issues such as smile line and incisor display [12]. In general, treatment includes surgical and non-surgical treatments. Non-surgical treatments for deep bite correction depending on the patients’ circumstances include one or a combination of the following treatments: changing the horizontal to vertical growth pattern with clockwise rotation of the mandible in growing short face patients, which causes an increase in the height of the lower anterior of the face, the intrusion of incisors in patients with high dental display, a labial forward tip of anterior teeth in bimaxillary retrusion patients, and the extrusion of molars [9,13].

There are different appliances and methods to correct deep bite orthodontics; extra-oral appliances such as j hook headgear are effective for controlling anchorage, although it requires the patient’s cooperation to achieve the desired result. One of the advantages of this device is that it is easy use by the patient and the dental team, and its disadvantages include the tendency to dental arch expansion [14,15].

**Figure 1:**
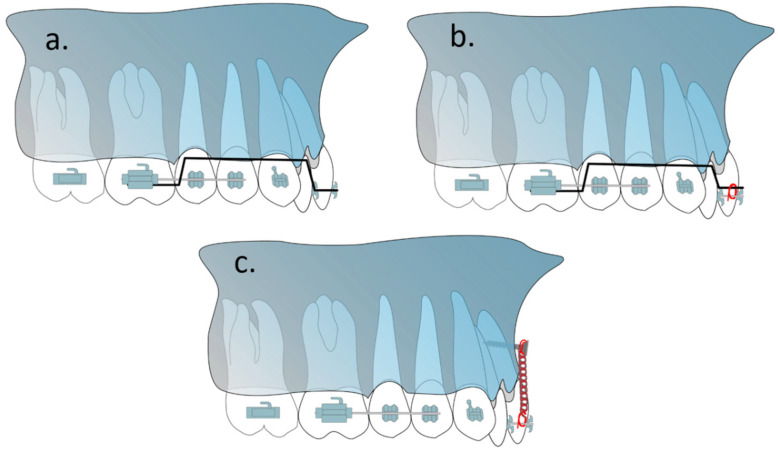
Different methods for intruding the anterior teeth (**a**) Rickett’s utility arch, (**b**) Burstone’s intrusion arch (also CTA), (**c**) mini-screw.

Intrusive arches such as Rickett’s utility arch, K-SIR loop, vertical loop, segmental intrusion arches (Burstone intrusion arch, Connecticut sion arch), and three-piece intrusion arch all use intraoral arches which use posterior teeth as an anchorage to intrude anterior teeth [16,17,18]. Rickett’s utility arch is inserted in the anterior teeth’s bracket slot while segmental intrusion arches usually become ligated to a base arch wire on the anterior teeth (Figure 1a,b). The segmental intrusion arches have lower impact on the anchorage teeth and have been shown to be far more effective for anterior teeth than utility arches which extrude anchorage teeth far more than other methods [19,20]. Burstone’s intrusion arch and Connecticut intrusion arch (CTA) both operate similarly but since CTA is prefabricated, it could help lower chairside time and because of its nickel–titanium construction, it needs fewer adjustment sessions [21].

Mini-screws are used as temporary anchorage devices (TAD) to produce various dental movements, including anterior retraction/canine retraction/distalization/molar uprighting/protraction [22,23,24,25,26]. Research has shown that mini-screws have the ability to apply force up to 500 g and at the same time remain intact until the end of the treatment [27]. The potential advantages of the TAD system are the possibility of applying force to the goal of tooth displacement in any direction, in the absence of mutually destructive forces, with no need for patient cooperation, and the possibility of changing the position and inclination of the upper and lower incisors to the desired state with the possibility of inserting incisors with minimal protrusion, higher intrusion speed, and no effect on posterior teeth [28,29,30] (Figure 1c).

Several different studies have reported conflicting results for the advantages of TADs compared to other common treatment methods for anterior teeth intrusion regarding the intrusion speed, the amount of true intrusion, the effects on incisor inclination and the effects on posterior teeth [30,31,32,33,34]. Because of this, we intend to investigate the effects of intrusion of anterior teeth by skeletal anchorage in deep bite patients in this systematic review and meta-analysis.

The PICOS (population, intervention, comparison, outcomes, and study design) for our study is defined as follows:

- P: Deep bite patients needing intrusion of anterior teeth with permanent dentition (14–50 years of age);
- I: Patients undergoing intrusion through the use of mini-implants (directly or indirectly),
C: Using other orthodontic methods of anterior tooth intrusion (i.e., utility arch, Connecticut intrusion arch);
- O: Overjet, overbite, cephalometric indicators (dental and skeletal), intrusion duration, intrusion rate and intrusion speed, root resorption;
- S: Clinical studies (cohort studies, RCTs and CCTs).

## 2. Materials and Methods

The present study is a systematic review and meta-analysis, which was conducted based on the PRISMA (Preferred Reporting Items for Systematic Reviews and Meta-Analyses) guidelines and Cochrane Handbook for Systematic Reviews of Interventions [35].

### 2.1. Eligibility Criteria

The inclusion criteria of our study are 1. studies that have investigated the intrusion of anterior teeth by means of mini-screws; 2. cohort studies and clinical trials; 3. human samples. The articles that were excluded from the study include 1. case–control studies and studies without a control group or no TAD group; 2. research with orthognathic surgery patients; 3. animal studies.

### 2.2. Review of Literature

In our systematic review study, in the first step, a systematic search strategy was designed using keywords related to the study topic. Then, using this strategy, a search was carried out for studies from the beginning to 15 September 2022 in PubMed, Scopus, Embase, Google Scholar, Web of Science, and Cochrane Central Register of Controlled Trials databases, and all the articles obtained were reviewed. The terms “mini-screw” and “intrusion” were used to further search grey literature sources for conference proceedings as well as Clinicaltrials.gov and WHO databases for protocols of ongoing studies. Additionally, a manual search of the references of included studies and the pertinent studies in the prominent orthodontic journals with IF >1 from 2008 to 2022 was conducted. These journals included *The Angle Orthodontist*, *American Journal of Orthodontics and Dentofacial Orthopedics*, *European Journal of Orthodontics*, and *Progress in Orthodontics*. The systematic search strategy for each database was unique and it is included in the Table 1.

### 2.3. Study Screening and Data Extraction

**Figure 2:**
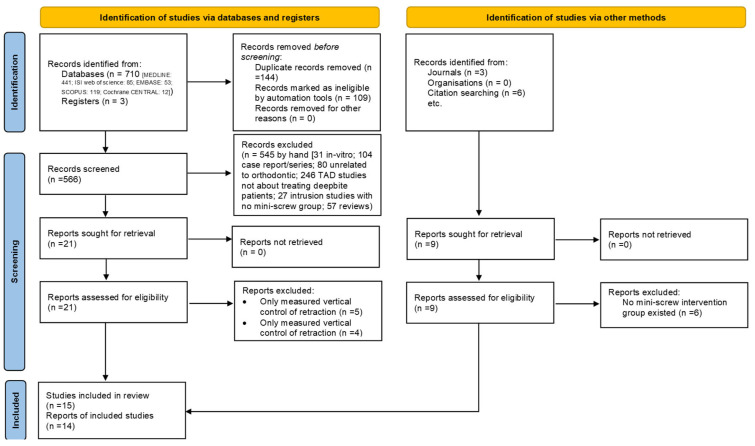
PRISMA flow diagram.

The title and abstract of related studies were examined by two researchers (GT, EB) separately and studies were excluded according to the inclusion and exclusion criteria. Any differences between these two researchers were resolved by the third researcher (HS). The researchers also evaluated the qualifying studies’ full-text versions. At this point, the study was excluded if it lacked relevant information or was unrelated to the review’s goals. The study selection is represented by the PRISMA flow chart in Figure 2.

The data of the selected articles were extracted by one researcher (AR) and the accuracy of the data extraction was checked by another researcher (EB). The desired information includes the names of the authors of the study, the countries in which the studies were conducted, the year of publication of the articles, the number of patients in the treatment and control groups, the average age of the patients, the gender of the participants in the studies, the duration of the study, and the inclusion and exclusion criteria of the study. The type of malocclusion, number and size of mini-screws, location of mini-screws, force, type of evaluation, and reported results for overjet, overbite, cephalometric indices, duration and rate of intrusion, and rate of root resorption were extracted from the included studies. The summary of the data related to the eligible studies is presented in Table 2 and in Table S1 of Supplementary Materials.

### 2.4. Quality Assessment

**Figure 3:**
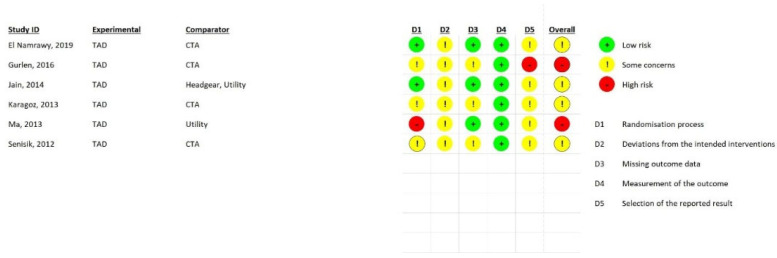
Results of RoB 2.0 risk of bias assessment.

**Figure 4:**
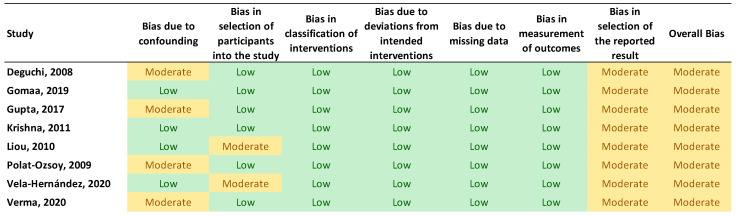
Results of ROBINS-I risk of bias assessment.

Two reviewers (E.B. and NK) independently evaluated the included studies’ quality using the Cochrane’s Risk of Bias in Non-randomized Studies of Interventions (ROBINS-I) tool and the Cochrane’s Risk of Bias Tool for Randomized Clinical Trials (RoB 2.0). The following seven domains are part of Cochrane’s risk of bias assessment for non-randomized studies: (1) bias brought on by confounding variables, (2) bias in participant selection, (3) bias in classification of interventions, (4) bias brought on by deviations from intended interventions, (5) bias brought on by missing data, (6) bias in outcome measurement, and (7) bias in the choice of the results that are reported. Each domain was classified as having a low, moderate, significant, or critical risk of bias based on the criteria. If a study obtained a moderate, serious, or critical risk ranking in any one of the seven categories, it was considered to have a moderate, serious, or critical risk for bias. Furthermore, the RoB 2.0 questionnaire has 5 domains, which include risks randomization process, deviations from the intended interventions, missing outcome data, measurements of the outcome, selection of the reported results, and in response to each domain, one of the three options of low risk, some concerns, and high risk, was used. The results of the RoB 2.0 and ROBINS-I are depicted in the Figure 3 and Figure 4.

In addition, the quality and reliability of the evidence and meta-analysis results were evaluated using the Grading of Recommendations Assessment, Development and Evaluation ranking system (GRADE). The GRADE system evaluates and classifies the quality and reliability of evidence according to the type of articles (randomized, non-randomized), risk of bias, risk of non-uniformity of results, indirectness of evidence (the measured variable is not related to the objective), inaccuracy in the results (high probability of error in measuring the results), and other cases (printing bias, high difference between two groups, outcome dependent on intervention dose, presence of confounding variable). The quality and confidence of the evidence is classified into four categories of high, medium, low, and very low confidence. For example, if there is low confidence, the results of that meta-analysis can be extended to clinical conditions with low confidence. The summary of the quality of the results of the landmark analysis before and after treatment by GRADE classification is shown in Table 3.

### 2.5. Statistical Analysis

Due to the continuous nature of all investigated variables (overjet, overbite, cephalometric indices), random-effects inverse variance meta-analysis was performed to evaluate positive outcomes reported by patients for different intrusion methods by mean difference (MD) at 95% confidence interval (95% CI). Meta-analysis was performed for dental examination (overjet and overbite), dental cephalometric, and skeletal cephalometric due to the similarity of the examinations in the studies. Due to the differences between the studies and the studied groups, a meta-analysis was not able to be performed for the length of time required for intrusion, the amount of intrusion per month, and root analysis. Cochran’s Q test was used to assess heterogeneity between studies and the I2 test was used to measure the degree of non-continuity in pooled calculations due to heterogeneity between studies. I2 values less than 30% indicate low heterogeneity, values between 30% and 60% indicate mean heterogeneity, and values above 60% are considered significant heterogeneity. If the heterogeneity between the studies was high, we tried to examine the results of the studies more homogeneously by using subgroup analysis and sensitivity analysis, which separates the studies with different comparison groups and the results outside the normal range.

The numbers reported in the articles related to the case and control groups were extracted and the confidence interval and mean difference were calculated using the Review Manager 5.4 software (Copenhagen, Denmark). A *p*-value of 0.05 was considered significant for hypothesis testing, but for heterogeneity, 0.01 was used due to low power. Because more than 10 studies were included in a couple of the meta-analyses, a visual inspection of the funnel plot was also used to gauge the publication bias of the included outcome measures.

## 3. Results

### 3.1. Study Selection and Characteristics

A total number of 710 articles were found through searching databases (MEDLINE: 441; Web of Science: 85; EMBASE: 53; SCOPUS: 119; Cochrane CENTRAL: 12) and hand searching related to the subject of the study. After removing 144 duplicate articles, the title and abstract of the remaining 566 articles were reviewed. Of these, 545 were excluded because they did not meet the inclusion criteria of our study (31 animal and in vitro studies, 104 case reports or case series, 80 studies not related to orthodontics, 246 mini-screw studies not related to the treatment of non-deep bite patients, 27 studies on anterior intrusion which lacked the mini-screw group, and 57 review studies) were excluded. The full text of the remaining 30 studies (21 studies by database search and 9 studies after manual search) were retrieved and analyzed, 5 of them because only the amount of vertical control of teeth during retraction was assessed and 10 of them were excluded because they did not have a mini-screw group. A total of 15 articles were included in our study for systematic review [15,29,30,31,32,33,34,36,37,38,39,40,41,42], and after data extraction, meta-analyses were performed on 14 studies, and for one study [42], only a systematic review was performed due to differences in outcomes and retainers under review with the rest of the studies.

Among all the articles, 14 studies were selected for meta-analysis, which were carried out between 2008 and 2020. In Table 2, the summary characteristics of the patients and the interventions that have been performed for them are summarized.

### 3.2. Risk of Bias

Figure 3 and Figure 4 show a summary of the risk of bias in the included studies. For randomized clinical trials, four studies had an unknown risk of bias while the two studies for Gurlen et al. and Ma et al. [37,39] had a high risk of bias. For the non-randomized studies, all of them were judged to have a moderate risk of bias mostly because of bias due to confounding factors, bias in selection of participants into the study, and bias in selection of the reported results. The GRADE evaluation method for evidence and certainty of outcomes revealed that the evidence for change in overbite and overjet between TAD and other anterior intrusion systems was of moderate quality. The serious inconsistency of results was the basis for the medium certainty assessment. Table 3 displays the results of the GRADE ranking.

### 3.3. Data Synthesis

Random-effects meta-analysis was performed for the outcome measures of dental indices (overbite, overjet), skeletal cephalometric indices and dental cephalometric indices. Additionally, the difference between the measurements of before and after intrusion treatment was used to check the effectiveness of different treatment methods. For abbreviations, all groups of segmental arch intrusions (Connecticut intrusion arch, Burstone intrusion arch, manual segmental arch) are called CTA in Forrest plot diagrams.

### 3.4. A Meta-Analysis of Dental Indices

**Figure 5:**
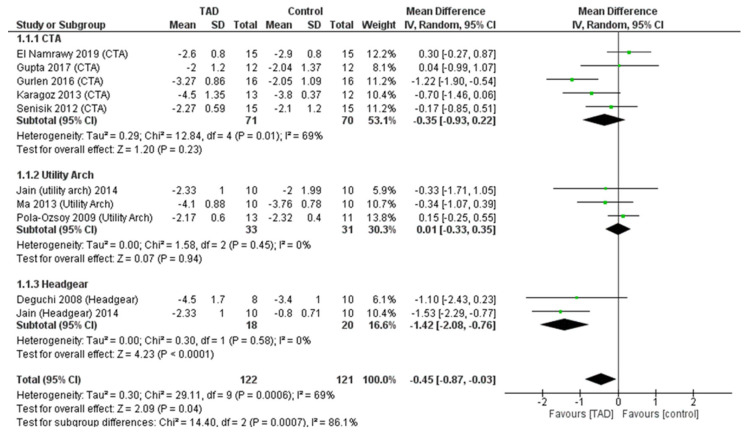
Forrest plot for random-effects analysis of differences in overbite before and after intrusion.

In the meta-analysis of the overjet after intrusion treatment, 10 studies and 243 patients (122 patients treated with TAD and 121 patients treated with other methods) were examined, and it was observed that the TAD groups had the ability to change the overjet more than the other groups (CTA, utility arch, headgear). In patients who used TAD for intrusion, an average of 0.45 mm reduction in overbite was observed compared to other patients (MD = −0.45, CI95% = −0.87, −0.03; *p* = 0.04) and this was statistically significant. However, the difference in overbite correction (0.45 mm) does not appear to be clinically significant. In doing this analysis, relatively high heterogeneity (I2 = 69%) was observed between the studies, which can be due to the difference between the comparison groups of studies. Considering this issue, a subgroup analysis was performed according to the interventions of the studies, and it was shown that both CTA and utility arch did not differ from TADs regarding the amount of overbite change. All differences between the control groups with TAD were due to the differences between mini-screws and J hook headgears (MD = −1.42, CI95% = −2.08, −0.76; *p* < 0.0001) and this difference is statistically and clinically significant. This issue is also confirmed by examining the test for subgroup differences (*p* = 0.0007) (Figure 5).

**Figure 6:**
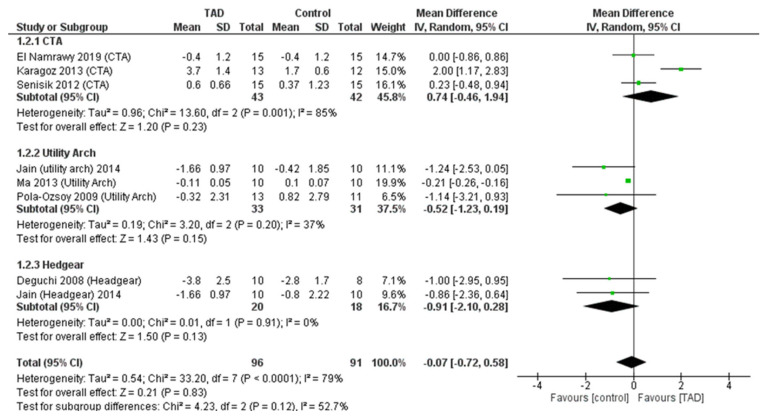
Forrest plot for random-effects analysis of differences in overjet before and after intrusion.

In a meta-analysis of overjet change after intrusion, eight studies including 187 patients (96 patients treated with TAD and 91 patients treated with other methods) were examined, and it was observed that there was no difference between the use of mini-screws and other treatment methods (MD = −0.91, CI95% = −2.10, 0.28; *p* = 0.83). This lack of difference existed regardless of the comparison group (CTA, utility arch, headgear). In the test for subgroup differences, there was no difference between the outcome of TAD compared to different comparison groups (*p* = 0.12) (Figure 6).

### 3.5. Meta-Analysis of Skeletal Cephalometric Indices

**Figure 7:**
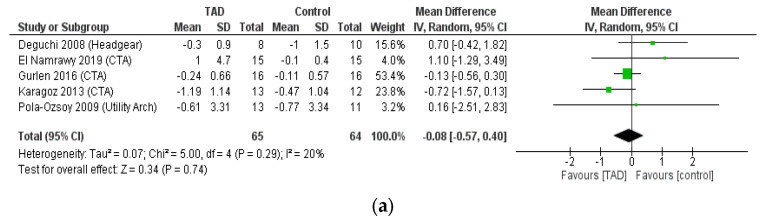

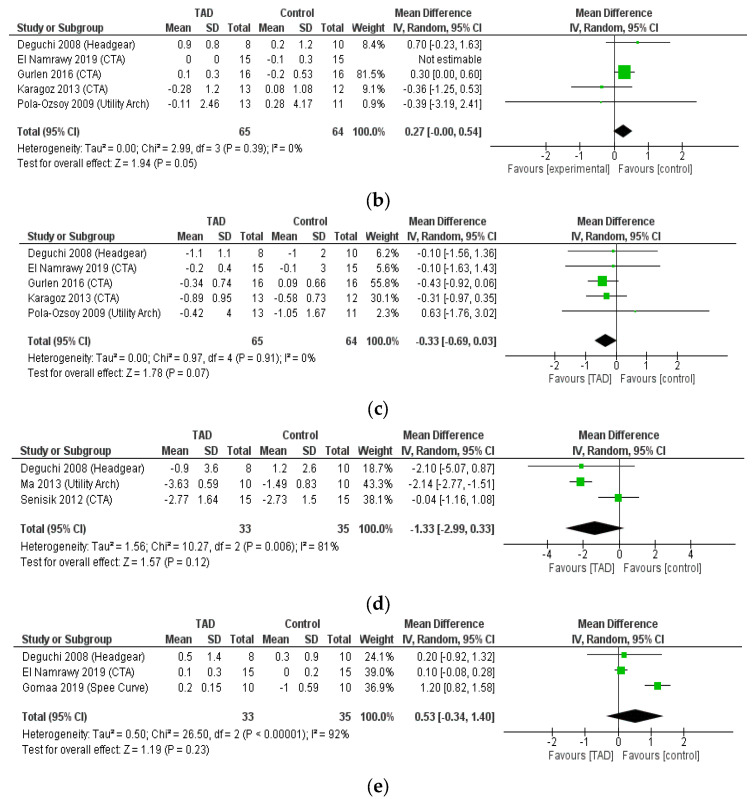
Forrest plot for random-effects analysis of differences in (**a**) SNA, (**b**) SNB, (**c**) ANB, (**d**) palatal plane, (**e**) occlusal plane changes before and after intrusion.

When examining SNA, SNB, and ANB angles, no difference was observed in terms of changes before and after treatment between the use of mini-screws and other treatment methods, which is expected. The result of a meta-analysis of SNA change after intrusion, after reviewing five studies on 129 patients was MD = −0.08, CI95% = −0.57, 0.4; *p* = 0.29. The result of a meta-analysis of SNB change after intrusion after reviewing five studies on 129 patients was also MD = 0.27, CI95% = −0.00, 0.54; *p* = 0.05. Finally, the result of a meta-analysis reviewing five studies on 129 patients, for ANB was MD = −0.33, CI95% = −0.69, 0.03; *p* = 0.07. Because of the small number of studies in these analyses, we had no ability to perform subgroup analyses. The noteworthy point in these three analyses was the low heterogeneity of the results between the studies (I2 < 20%), which indicates the similarity of the results of these independent studies of the type of treatment investigated (Figure 7).

In addition, in the examination of changes in the occlusal plane and palatal plane, no significant difference was observed between different treatment methods (TAD, CTA, utility arch, headgear, speed curve). The result of meta-analysis of three studies including 68 patients for occlusal plan and palatal plan was MD = −1.33, CI95% = −2.99, 0.033; *p* = 0.12 and MD = 0.53, CI95% = −0.34, 1.4; *p* = 0.23, respectively. The significant issue here was the high degree of heterogeneity of the results of the studies in these two analyses (I2 > 80%) and since there was no ability to perform subgroup analysis due to the small number of studies, the results of these meta-analyses should be considered with caution (Figure 7).

**Figure 8:**
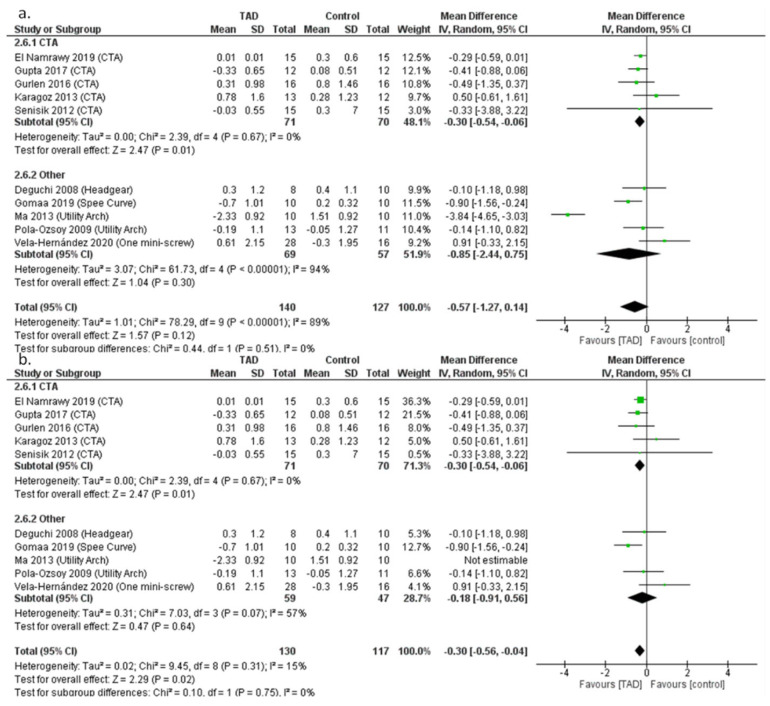
Forrest plot for random-effects analysis of differences in mandibular plane changes (**a**) with Ma et al. study, (**b**). without Ma et al. study before and after intrusion.

For mandibular plane changes, no significant difference was observed after reviewing 10 studies with 267 patients (MD = −0.85, CI95% = −2.44, 0.75; *p* = 0.51) (Figure 8a). The degree of heterogeneity in this meta-analysis was very high (I2 = 89%), which could be due to the differences in the treatment method between the comparison groups, or it could be due to the large difference in the results of the study by Ma et al. with the rest of the studies. After performing a sensitivity analysis and removing the study by Ma et al., the degree of heterogeneity of the study results reached 15%, which shows the effect of the Ma study on heterogeneity. In addition, after removing this study, the results of the meta-analysis changed and a statistically significant difference was observed between the mandibular plane change after mini-screw treatment and other treatment methods (MD = −0.30, CI95% = −0.56, −0.04; *p* = 0.02) (Figure 8b). Furthermore, after performing subgroup analysis and separating studies with CTA group and other comparison groups, the difference between mini-screw compared to CTA was statistically significant, but clinically not very large. The mandibular plane was reduced by 0.3 degrees (MD= −0.30, CI95% = −0.54, −0.06; *p* = 0.01). However, no significant difference between the TAD groups and other comparison groups with the Ma et al. study (MD = −0.85, CI95% = −2.44, 0.75; *p* = 0.3) or without that study (MD = −0.18, CI95% = −0.91, 0.56; *p* = 0.64) was observed.

### 3.6. Meta-Analysis of Dental Cephalometric Indices

**Figure 9:**
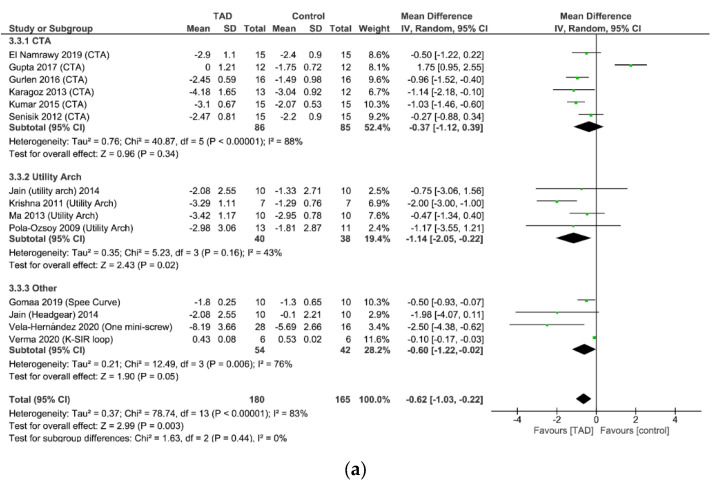

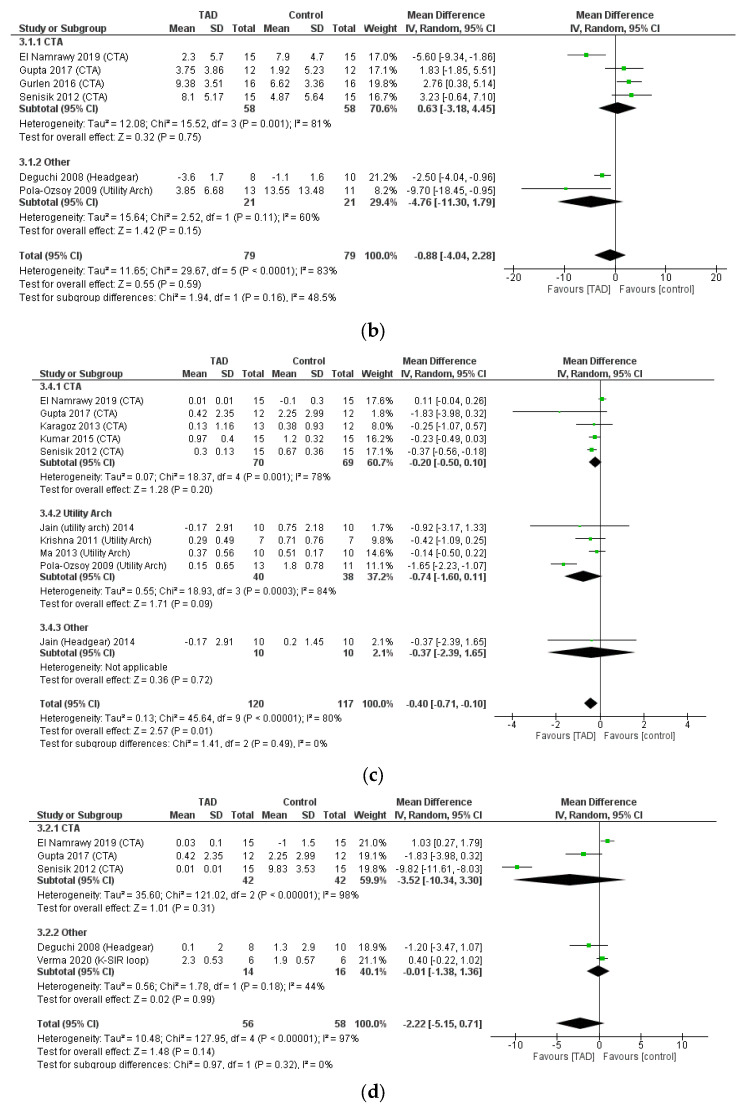

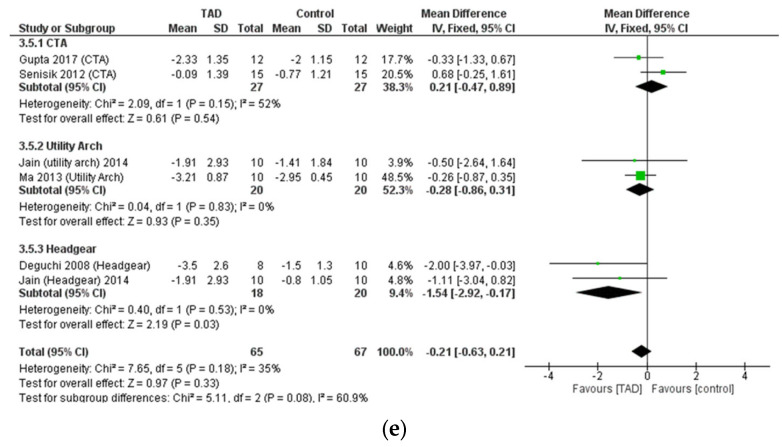
(**a**). Forrest plot for random-effects analysis of differences in U1-PP distance. (**b**) Forrest plot for random-effects analysis of differences in a. U1-PP angle. (**c**) Forrest plot for random-effects analysis of differences in a. U6-PP distance. (**d**) Forrest plot for random-effects analysis of differences in a. U6-PP angle. (**e**) Forrest plot for random-effects analysis of differences in a. U1-stm distance.

In examining the U1-PP distance (the change in the distance between the CR of the central incisor teeth and the palatal plane), which indicates the amount of true intrusion of the teeth, the mini-screw group on average had 0.62 mm more change than the other groups. The result of this meta-analysis is based on the analysis of 13 studies with 301 patients (152 patients treated with mini-screws and 149 patients treated with other methods) (MD = −0.62, CI95% = −1.03, −0.22; *p* = 0.003). Due to the high heterogeneity of the studies in this analysis (I2 = 83%), a subgroup analysis was performed by separating the studies of CTA, utility arch, and other treatments (spee curve, headgear, K-SIR loop, one mini-screw). U1-PP change was not significantly different between mini-screw and CTA groups (MD = −0.37, CI95% = −1.12, 0.39; *p* = 0.34), while the difference between groups for mini-screw and utility arch (MD =−1.14, CI95% = −2.05, −0.22; *p* = 0.02) as well as mini-screw and other treatments (MD = −0.60, CI95% = −1.22, −0.02; *p* = 0.05) was significant. This means that the use of mini-screws causes an average of 1.14 mm more real intrusion than the utility arch (Figure 9a).

In examining the change in the U1-PP angle (the angle of the central incisor tooth with the palatal plane), which indicates the degree of tipping of the incisor tooth during its intrusion, there was no significant difference between the change in this angle between intrusion by mini-screw and other methods (MD = −0.88, CI95% = −4.42, 2.28; *p* = 0.59). In the subgroup analysis, no difference between mini-screw and CTA (MD = 0.63, CI95% = −3.18, 4.45; *p* = 0.75), as well as mini-screw and other treatments (utility arch, headgear) (MD = −4.76, CI95 % = −11.30, 1.79; *p* = 0.15) was seen either. This shows that mini-screws and other treatment methods work similarly in terms of tipping the incisor teeth and producing the same amount of tipping (Figure 9b).

Moreover, when examining the change in the U6-PP distance (the distance between the tip of the mesiolbuccal cusp of the upper first molar and the palatal plane) after intrusion, the patients in the mini-screw group had a smaller distance change than the other treatment groups (MD = −0.40, CI95% = −0.71, −0.10; *p* = 0.01). This difference is statistically significant and means that maxillary molar teeth are extruded 0.4 mm less than other treatments when using mini-screws. This meta-analysis has high heterogeneity (I2 = 80%) and after subgroup analysis, it was found that the difference in U6-PP distance change for CTA, utility arch and headgear groups was not significantly different (Figure 9c).

There was no significant difference between the use of mini-screws and the use of other methods (CTA, K-SIR loop, headgear) regarding the angle of the upper first molar teeth with palatal plane (U6-PP), whether in the main analysis (MD = −2.22, CI95% = −5.15, 0.71; *p* = 0.14) or subgroup analyses. The significant problem with this analysis was the high degree of heterogeneity (I2 = 97%) of the results of these studies, which makes it difficult to rely on these results (Figure 9d).

Finally, in the examination of the change in the distance between the central edge of the maxilla and the upper lip (U1-stm) after examining six studies with 132 patients, no significant difference was found between the use of mini-screws and other treatment methods (MD = −0.21, CI95% = −0.63, 0.21; *p* = 0.33). However, after subgroup analysis, the use of mini-screws significantly changes the distance more than the use of J hook headgears (MD = −1.54, CI95% = −2.92, −0.17; *p* = 0.03). This result means that the mini-screw can reduce the incisal projection at res by an average of 1.54 mm more than the headgear (Figure 9e).

**Figure 10:**
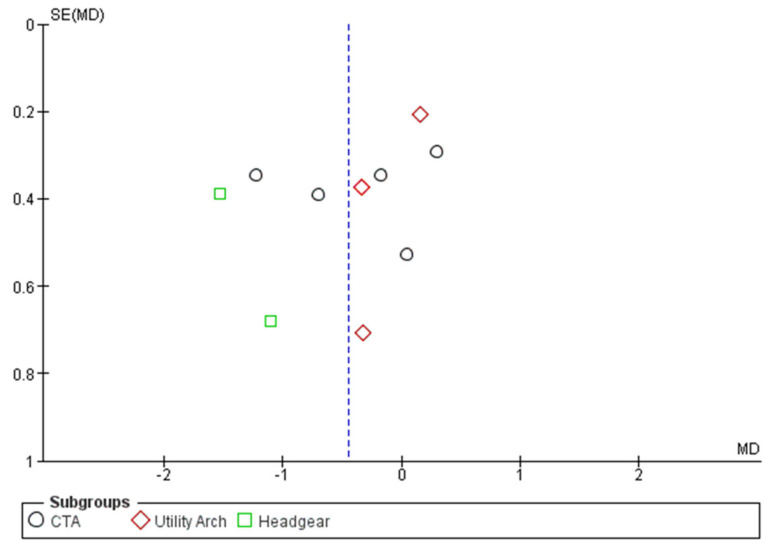
Funnel plot for random-effects analysis of differences in overbite before and after intrusion for evaluation of publication bias.

To check publication bias, the meta-analysis funnel plot for overbite change was visually examined. According to the appearance of this funnel plot, the existence of publication bias among these studies is rejected (Figure 10).

## 4. Discussion

### 4.1. Summary of Evidence

In our study, a meta-analysis was performed for dental indices, skeletal cephalometric indices, and dental cephalometric indices. Regarding overbite change, performing intrusion by the TAD method showed a 0.45 mm reduction in overbite more than performing intrusion with other treatment methods which was statistically significant but not substantial clinically. Furthermore, by examining the subgroups, it seems that the mini-screw caused more intrusion only compared to headgear, and there is no significant difference with utility arch and CTA in this regard. Since in most patients, intrusion by the clinician continued until after achieving an acceptable overbite, the closeness of the results of different treatment methods on the teeth can be expected.

For overjet change, no significant difference has been observed between different treatment methods, due to the similar point of application for force (between laterals and canines) when using a J hook, TAD, and CTA. Additionally, due to the difference for the point of force application in the utility arch, it is expected that the amount of change in overjet would be more positive in these groups. Although this issue was also observed in the meta-analysis (MD = −0.51), this difference was not statistically significant.

Finally, for the study by Gomma et al. which was not included in these meta-analyses, in the control group, the accentuated curve of spee was used in the upper jaw to reduce overbite and to intrude anterior teeth. In this study, it was observed that the use of accentuated curve of spee and mini-screw did not have a significant difference in terms of overbite change (MD = −0.1 mm), but in terms of overjet change, the use of TAD resulted in more overjet reduction (MD = −2.5 mm) and this difference was significant [29].

When examining SNA, SNB, ANB, and palatal plane, no significant difference was observed between different treatment groups, which is expected since most of the changes in intrusion interventions affect the teeth. While based on the differences in molar extrusion, it was expected that the change in the occlusal plan and the mandibular plan would be different in different intrusion methods; however, in our meta-analyses, it was found that the change in the occlusal plane was not significantly different when using mini-screw and other methods, and the mandibular plane showed only a slight difference (0.3 degrees) between mini-screw and CTA.

To check the amount of real intrusion, the change in the CR distance of the central incisor and palatal plan was examined and it was observed that mini-screws and CTA did not significantly differ in terms of the amount of real intrusion, while mini-screws had significantly more real intrusion than utility arch (1.14 mm on average) and other treatments (0.6 mm on average).

Regarding incisor forward tipping (U1-PP angle) during intrusion, no difference between mini-screws and other treatment methods could be found. It is possible that the use of rectangular wire in the slots of the brackets when using the utility arch compensates the side effects of the force applied in front of the CR of the incisor teeth.

However, when comparing the effect of intrusion with mini-screws versus other treatment methods, it seems that the use of mini-screws causes less extrusion of maxillary first molar teeth (change in U6-PP distance) compared to other treatments. This is despite the fact that during the subgroup analysis, no significant difference is seen in the two-by-two comparison of mini-screw with CTA, utility arch, and other treatments. This issue could be due to the increase in the power of the main meta-analysis with higher number of studies and the number of participants, which has reduced the standard error of the analysis and made the results of the meta-analysis more meaningful when considering the overall treatments [43]. Furthermore, no significant difference was seen in comparing the upper molar tooth tip during treatment with mini-screw and various other methods.

Finally, the mini-screw showed a significant reduction (1.54 mm) in incisor display at rest with J hook headgear, but this difference was insignificant for the CTA or utility arch. This result is in agreement with the result of the overbite change, which shows that the use of headgear is less effective than mini-screws, at least in terms of reducing the appearance of overbite and incisor display.

### 4.2. Other Considerations

Regarding the duration required for intrusion, we were not able to perform a meta-analysis due to differences in measurement methods between studies. In the studies of Kumar et al., Nayak et al., and Jain et al., instead of examining intrusion until an acceptable overbite was obtained, the duration of intrusion was fixed for both TAD and comparison groups (six months for Nayak et al. and Kumar et al. and four months for Jain et al.), and the actual intrusion rate in this period was instead calculated [32,38,41]. Meanwhile, the rest of the intrusion studies continued until the end of obtaining an acceptable overbite and the time required to obtain this ideal overbite was investigated. In all these studies except the study by El Namrawy et al. [31], the time required for intrusion in the TAD group was less or equal to the comparison groups, while in the study by El Namrawy et al., the time required to obtain a suitable overbite was non-significantly higher for the mini-screw group. Regarding the rate of intrusion, in two studies the rate of intrusion in the TAD group compared with comparison groups was significantly higher (Gupta et al., Polat -Ozsoy et al.), and in two studies, it was slightly higher (Senisik et al., Verma et al.) [30,33,34,36].

Regarding root resorption, in the evaluated studies, there was no significant difference between the groups in terms of the amount of root resorption, both linearly (Deguchi et al., Karagoz et al., Vela Hernandez et al.) [15,40,44] and volumetrically (Liou et al.) [42]. In all investigated intrusion groups, moderate root resorption in the apex (about 1 to 2 mm) was observed. Furthermore, in the review study by Martin et al. in human and animal studies, a moderate amount of root external resorption was seen after intrusion using TADs [45]. Therefore, the root characteristics of anterior teeth should be considered before the initiation of intrusion treatment by any device, including TADs. A history of trauma to the anterior teeth or previous root canal treatment, and the presence of certain hormonal problems could be considered risk factors for root resorption, and intrusion should be avoided in these cases [45,46].

Regarding the characteristics of the force applied to the teeth, different force magnitudes (from 50 g to 100 g) were used in different studies for anterior intrusion. According to the study by Steenbergen et al., increasing the amount of intrusive force from 40 to 80 g does not change the rate of intrusion or the amount of tooth tipping, but increases the probability of root resorption [47]. Since intrusive forces are applied to a small point (at or near the apex), too much force can cause unwanted side effects. Burstone et al. also suggested using 50 g of force for the intrusion of the maxillary incisors, and since all the studies included in our review examined the intrusion of the four maxillary incisors, the 50 gr figure seems appropriate. Another issue is that in terms of the stability of the magnitude of applied force, it seems that the use of a mini-screw with Ni-Ti close coil spring could provide the most stable force. According to Burstone et al., force stability in the optimal force zone can create optimal intrusion without delays caused by bone ischemia. High force stability is also seen in wires with low deflection rates such as CNA used in CTA [48].

Regarding the placement of mini-screws, some studies have chosen the point between the central and lateral roots, while others have chosen the point between the lateral and canine roots, and even the midline point (one mini-screw). According to biomechanical studies, placing the mini-screw in the more posterior area (between lateral and canine) causes the forces line of action to pass near the CR of the incisor teeth and could lead to less tipping during intrusion [49]. In our review, after performing a subgroup analysis on the upper incisal tip (U1-PP), no significant difference was seen between placing mini-screws between lateral and canine or between central and canine (*p* = 0.32). Additionally, in the study by Vela-Hernandez et al., no significant difference in incisal inclination was found when one mini-screw in the midline or two mini-screws between the lateral and canine were used [40].

Based on these findings, we can conclude that most anterior intrusion approaches achieved the intended overbite correction during their interventions and were at least aesthetically successful in treating deep bite patients. Different treatment methods also showed similar root resorption and this should be an important consideration for patient selection. The fundamental difference between mini-screws and other treatments appears to be the absence of undesirable biomechanical effects (incisor tipping, molar extrusion) and the higher true intrusion achieved with this approach.

Finally, in our review, the differences before and after intrusion (change scores) were used for meta-analysis. According to the Cochrane’s Handbook, the results of the difference between before and after treatment can be included in the meta-analysis just like the outcome measures after the treatment, and since the difference between before and after the treatment has less variance than the after-treatment outcome measures by themselves, the strength of the meta-analysis using these data seems to be higher [35].

### 4.3. Comparison with Other Systematic Reviews

Two other systematic review studies also investigated the effect of using mini-screws in intrusion. In the first study by Atalla et al., the use of mini-screws in anterior intrusion had a significantly higher amount of intrusion than the conventional segmented arch group (MD = 0.78, CI95% = 0.28–1.29), which is inconsistent with the result of our study. It should be noted that in the Atalla et al. review, studies by Nayak et al. and Jain et al. [38,41] which used utility arch were counted as segmented arch studies and were included in the meta-analysis. In the Atalla et al. systematic review, six studies were included, and in each meta-analysis, a maximum of three studies were used, while in our meta-analysis, about 14 studies were included to evaluate the amount of intrusion, six of which used segmented arch (CTA). Moreover, in the study by Atalla et al., the amount of incisor and molar tipping caused by mini-screws was not different from segmented arch, but the rate of anchorage loss (molar extrusion) in the segmented arch group was higher than that of mini-screws, and all these results are consistent with our study [50].

In another review study by Sosly et al. [51], seven RCT studies were examined, and by conducting a meta-analysis, the rate of overbite reduction and true intrusion of incisors was significantly higher in the mini-screw group than in the utility arch and CTA groups, which is different from our study. However, the amount of molar extrusion in the mini-screw group was significantly lower, which is consistent with the results of our meta-analysis. Again, the difference between the results of our study and the meta-analysis of Sosly et al. could be because of the number of included studies (at most, five studies in Sosly’s meta-analysis vs. 14 in our meta-analyses). Furthermore, in the Sosly et al. study, due to the small number of studies, it was not possible to perform subgroup analysis to investigate the differences between different comparative groups (CTA, utility arch) [51].

### 4.4. Implications for Clinical Setting

Orthodontic deep bite treatment is a form of biomimetic orthodontic treatment. By using mini-screws, headgears, CTAs, or utility arches, clinicians strive to restore the normal vertical growth balance of anterior and posterior teeth by preventing incisor overeruption and molar undergrowth. Further, the intrusion of anterior teeth and/or the extrusion of posterior teeth mimics the growth pattern of a vertical grower, altering the facial height of a patient suffering from short facial height.

According to the results of our study, it can be concluded that the use of mini-screws, while they may not have much superiority in the amount of intrusion of incisors compared to the use of CTA, are advantageous regarding controlling the extrusion of molar teeth and intrusion rate. Moreover, utility arches and headgears seem to be not suitable for intruding incisors. Finally, the intrusion of anterior teeth can be associated with root resorption, regardless of the method, and therefore the patient should be carefully evaluated before the initiation of intrusion, and the use of heavy forces should be avoided in intrusion patients.

### 4.5. Strengths and Limitations

One of the strengths of this study was the in-depth electronic and manual search of studies, as well as the subgroup analysis and sensitivity analysis performed on the results of meta-analyses to minimize the sources of heterogeneity from the results. In addition, the quality of evidence was evaluated using GRADE (Grading of Recommendations, Assessment, Development and Evaluations) which showed moderate certainty regarding the results of meta-analysis.

Among the weak points of our study included the high heterogeneity in the meta-analyses and the differences between the methods of performing intrusions (location of mini-screws, the amount of force applied, alignment before intrusion). Furthermore, the small number of patients in the base studies was another weakness of this meta-analysis, and it is suggested that RCT studies with a higher number of participants be conducted to solve these issues.

## 5. Conclusions

The use of mini-screws leads to lower overbite and higher true intrusion (about 0.45 and 0.62 mm, respectively) compared to the use of other methods for intruding upper incisors. Furthermore, the effect of TAD on the extrusion of molar teeth is significantly less (by 0.4 mm) than other methods. Meanwhile, the use of mini-screws shows no significant difference in terms of incisor and molar inclination, changes in skeletal angles, and root resorption compared to other treatment methods.

When comparing mini-screws and segmented intrusion arches (Connecticut intrusion arch, Burstone), there were no significant differences between them in terms of reduction of overbite and true intrusion, but mini-screws performed better in terms of anchorage preservation and intrusion rate.

Finally, all intrusion methods could cause root resorption in anterior teeth, and therefore the clinician must pay attention to the incisor characteristics during case selection.

## Figures and Tables

**Table 1:** Databases. Applied search strategy, and numbers of retrieved studies.

Database of Published Trials, Dissertations, and Conference Proceedings	Search Strategy Used	Hits
MEDLINE searched via PubMed searched on 12 September 2022, via www.ncbi.nlm.nih.gov/sites	#1 TAD OR temporary anchorage device OR mini?screw OR micro?implant OR mini?plate OR titanium plate OR surgical plate OR skeletal anchorage 58,286 #2 anterior impaction OR incisor intrusion OR incisal intrusion OR deep?bite OR deep overbite OR gummy smile OR short face OR anterior intrusion 18,346 #3 #1 AND #2 434	441
Web of Science Core Collection was searched via Web of Knowledge on 12 September 2022, via apps.webofknowledge.com	#1 TS = (TAD OR temporary anchorage device OR mini?screw OR #2 micro implant OR surgical miniplate OR mini?plate) 14,669 TS = (anterior intrusion OR incisal intrusion OR deep?bite OR short face) 37,935 #3 #1 AND #2 85	85
EMBASE searched via Ovid on 13 September 2022, via http://ovidsp.dc2.ovid.com	(tad OR ‘temporary anchorage device’/exp OR ‘temporary anchorage device’ OR ‘miniscrew’/exp OR ‘miniscrew’ OR ‘miniplate’/exp OR ‘miniplate’) AND (((‘overbite’/exp OR ‘overbite’ OR ‘incisor’/exp OR incisor) AND (‘intrusion’/exp OR intrusion) OR incisal) AND (‘intrusion’/exp OR intrusion) OR short?face)	53
Scopus searched via Scopus on 10 September 2022, via https://www.scopus.com	TITLE-ABS-KEY(TAD OR temporary anchorage device OR mini?plate OR mini?screw OR skeletal anchorage) AND ALL(deep?bite OR incis* intrusion OR short?face)	119
Cochrane Central Register of Controlled Trials searched via the Cochrane Library Searched on 15 September 2022, via www.thecochranelibrary.com	#1→TAD→274 #2→temporary anchorage device→23 #3→mini?plate→121 #4→mini?screw→134 #5→deep?bite→10 #6→anterior intrusion→65 #7→incisal intrusion→6 #8→incisor intrusion→49 #9→(#1 OR #2 OR #3 OR #4) AND (#5 OR #6 OR #7 OR #8)→12	12
Total		710

**Table 2:** Summary characteristics of included studies.

Author Year	Study Design	Sample Size	Gender	Age	Malocclusion	Number of Mini-Screws	Mini-Screw Placement	Force	Assessment	Group	Subjects
Deguchi 2008	Retro Cohort	18	2 M; 16 F	20.7 ± 2.5 (headgear); 21.5 ± 3.7 (mini-screw)	NR (probably Cl II)	2, 1.5 × 6 mm	Between central and lateral	100 gMS and HG	Root resorption; intrusion duration; cephalometric (angular; linear)	Mini-screw	8
										Headgear	10
El Namrawy, 2019	RCT	30	9 M; 21 F	15.3 ± 1 (mini-screw) 14.8 ± 1 (intrusive arch)	Class I or Class II	2, 1.4 × 6 mm	Between lateral and canine	100 gr	Pain; cast measurements (overbite; over jet; inter-canine width; inter-molar width); cephalometric (skeletal; dental; soft tissue)	Mini-screw	15
										Intrusive arch (segmental)	15
Gomaa, 2019	Retro cohort	20	2 M; 18 F	18–24	Class I or II	2, 1.3–1.6 × 6–8 mm	Between central and lateral	NR	Smile analysis; overbite; overjet; cephalometric	Mini-screw	10
										Curve of spee arch wire, both dentitions	10
Gupta, 2017	Prospective cohort	24	NR	17.75 ± 3.49 (mini-screw) 18.75 ± 3.47 (intrusive arch)	NR	2, 1.3 × 8 mm	Between lateral and canine	30 gr per side (60 gr)	Overbite; cephalometric (angular; linear)	Mini-screw	12
										Connecticut intrusion arch	12
Gurlen, 2016	RCT	32	16 M; 16 F	14.65	NR	2, 1.4 × 7 mm	Between central and lateral	60 gr for both	Cephalometric (dental; skeletal); root resorption	Mini-screw	16
										Connecticut intrusion arch	16
Jain, 2014	RCT	30	11 M; 19 F	16–22	NR	2, 1.4 × 6 mm	Between central and lateral	1.5 ounces per side (80 gr) for MS, UA; 2 for J hook	Overjet, overbite, cephalometric (dental)	Mini-screw	10
										Headgear	10
										Utility arch	10
Karagoz, 2013	RCT	25	11 M; 14 F	18.2 ± 3.3	NR	2, 1.4 × 8 mm	Between central and lateral	100 gr	Overjet, overbite, cephalometric (dental; skeletal); root resorption	Mini-screw	13
										Segmental + TPA	12
Nayak, 2011	Prospective cohort	14	NR	NR	NR	1, 2 × 8 mm	In the midline in the frenum region	50 gr	Cephalometric (dental)	Mini-implant	7
										Utility arch	7
Kumar, 2015	Prospective cohort	30	NR	15–20	Class II Div 1	2, 1.3 × 7 mm	Between central and lateral	60 gr for both	Cephalometric (dental)	Mini-screw	15
										Connecticut intrusion arch	15
Liou, 2010	Retro cohort	50	4 M; 46 F	25.4 ± 5.6	NR	2, 2 × 9 mm	Infrazygomatic	100 gr intrusive, 250 gr retractive	Root resorption	Mini-screw Anchorage	30
										Control	20
Ma, 2013	RCT	20	NR	22.41 ± 2.02	NR	2, 1.6 × 8 mm	Between central and lateral	50 gr	Overjet; overbite; cephalometric (dental; skeletal)	Mini-screw	10
										Utility arch	10
Polat-Ozsoy, 2009	Prospective cohort	24	10 M; 14 F	20.90 ± 7.12 (mini-screw) 15.25 ± 3.93 (utility arch)	NR	2, 1.2 × 6 mm	Between lateral and canine	50 gr mini-screw	Overjet; overbite; cephalometric (dental; skeletal)	Mini-screw	13
										Utility arch	11
Senisik, 2012	RCT	45	19 M; 26 F)	20.29 ± 3.12	Class II Division 2	2, 1.3 × 5 mm	Between lateral and canine	90 gr mini-screw; 60 gr CTA	Overjet; overbite; cephalometric (dental; skeletal)	Mini-screw	15
										Connecticut intrusion arch	15
										Control	15
Vela-Hernández, 2020	Retro cohort	44	20 M; 24 F	36.6 ± 4.9	Class I	1/2, both 1.6 × 8 mm	In midline/Between lateral and canine	90 gr per mini-screw	Overbite; cephalometric (dental; skeletal)	One mini-screw	16
										Two mini-Screws	28
Verma, 2020	Prospective cohort	12	NR	29.5 ± 2.1	Class I or Class II Div 1	2, 1.3 × 8 mm	Between central and lateral	30 gr each side	Cephalometric (dental)	Mini-screw	6
										K-SIR loop	6

Abbreviations: M: male; F: female; NR: not reported.

**Table 3:** The GRADE ranking of available evidence.

Certainty Assessment							№ of Patients		Effect		Certainty	Importance
№ of Studies	Study Design	Risk of Bias	Inconsistency	Indirectness	Imprecision	Other Considerations	TAD	Other Treatment Methods	Relative (95% CI)	Absolute (95% CI)		
Overbite												
10	Observational studies	Not serious	Serious ^a^	Not serious	Not serious	None	122	121	-	MD 0.45 lower (0.87 lower to 0.03 lower)	⨁⨁⨁◯ Moderate	
Overjet												
8	Observational studies	Not serious	Serious ^a^	Not serious	Not serious ^a^	None	96	91	-	MD 0.07 lower (0.72 lower to 0.58 higher)	⨁⨁⨁◯ Moderate	

CI: confidence interval; MD: mean difference. Explanations, ^a^. The test of heterogeneity for this outcome is high.

## Data Availability

The data that support the findings of this study (data collection forms; data extracted from included studies; data used for analyses) are available upon request from the corresponding authors, H.S. and A.R.

## Supplementary Materials

The following supporting information can be downloaded at: https://www.mdpi.com/article/10.3390/biomimetics8010101/s1, Table S1: The characteristics of the included studies.
