# Supplementary material for: The Effects of Intrusion of Anterior Teeth by Skeletal Anchorage in Deep Bite Patients; A Systematic Review and Meta-Analysis
Source: Biomimetics (Basel). 2023 Mar 2;8(1):101. doi: 10.3390/biomimetics8010101 (PMC10046359; doi:10.3390/biomimetics8010101)
Supplement: Supplementary file 1 [file biomimetics-08-00101-s001.zip › biomimetics-2164917-supplementary.pdf]

Author or Year	Study Design	Sample Size	Gender	Age	Malocclusion	Number of mini-screws	Mini-screw Placement	Force	Assessment	Group	Subjects	Outcomes (Pre-Post Differences)																																									
												Overbite		Overjet		SNA (°)		SNB (°)		ANB (°)		PP-SN (°)		Occl Pl (°)		MP-SN (°)		SN-U1 (°)		UL-E (mm)		LL-E (mm)		PP-U1 (°)		PP-U6 (°)		PP-U1 (mm)		PP-U6 (mm)		Stm-U1 (mm)		Duration		Root Resorption (mm)		Intrusion Rate (mm/month)		ANS-Me (mm)	
												M	SD	M	SD	M	SD	M	SD	M	SD	M	SD	M	SD	M	SD	M	SD	M	SD	M	SD	M	SD	M	SD	M	SD	M	SD	M	SD	M	SD	M	SD	M	SD	M	SD
Deguchi 2008	Retro Cohort	18	2M; 16F	20.7 ± 2.5 (headgear); 21.5 ± 3.7 (mini-screw)	NR (Probably Cl II)	2, 1.5 X 6 mm	Between central and lateral	100 gMS and HG	Root Resorption; Intrusion Duration; Cephalometric (Angular; Linear)	Mini-screw	8	-4.5	1.7	-3.8	2.5	-0.3	0.9	0.9	0.8	-1.1	1.1	0.5	1.4	-0.9	3.6	0.3	1.2	-6.2	8.9					-3.6	1.7	0.1	2.0					-3.5	2.6	6.6	0.7	0.8	0.4				
										Headgear	10	-3.4	1	-2.8	1.7	-1.0	1.5	0.2	1.2	-1.0	2.0	0.3	0.9	1.2	2.6	0.4	1.1	-8.5	9.8					-1.1	1.6	1.3	2.9					-1.5	1.3	7.3	1.6	1.2	0.4				
El Namrawy, 2019	RCT	30	9M; 21F	15.3 ± 1(mini-screw) 14.8 ± 1(intrusive arch)	Class I or Class II	2, 1.4 X 6 mm	Between lateral and canine	100 gr	Pain; Cast Measurements (Overbite; Overjet; Inter-canine width; Inter-molar width); Cephalometric (Skeletal; Dental; Soft Tissue)	Mini-screw	15	-2.6	0.8	-0.4	1.2	1	4.7	0	0	-0.2	0.4	0.1	0.3			0	0	2	5.5	-0.3	0.6	-0.1	0.3	2.3	5.7	0.03	0.1	-2.9	1.1	0	0			5.3	1			0.49	0.16		
										Intrusive arch (segmental)	15	-2.9	0.8	-0.4	1.2	-0.1	0.4	-0.1	0.3	-0.1	03	0	0			0.3	0.6	7.7	4.7	-0.06	0.7	-0.2	0.6	7.9	4.7	-1	1.5	-2.4	0.9	-0.1	0.3			4.8	1			0.6	0.16		
Gomaa, 2019	Retro Cohort	20	2M; 18F	18-24	Class I or II	2, 1.3-1.6 X 6-8 mm	Between central and lateral	NR	Smile Analysis; Overbite; Overjet; Cephalometric	Mini-Screw	10	-2.8	1.22	-2.8	2.23							0.2	0.15			-0.7	1.01											-1.8	0.25			-3.6	2.35								
										Curve of Spee Arch wire, both dentitions	10	-2.7	1.49	-0.3	0.86							-1	0.59			0.2	0.32											-1.3	0.65			-1.5	0.59								
Gupta, 2017	Prospective Cohort	24	NR	17.75 ± 3.49(mini-screw)	NR	2, 1.3 X 8 mm	Between lateral and canine	30 gr per side (60 gr)	Overbite; Cephalometric (Angul	Mini-Screw	12	−2.	1.2													−0.33	0.65	3.92	4.03					3.75	3.86	0.42	2.35	−2.46	1.21	0.42	2.35	−2.33	1.35	4.6	2.3			0.53	0.26		

Author or Year	Study Design	Sample Size	Gender	Age	Malocclusion	Number of mini-screws	Mini-screw Placement	Force	Assessment	Group	Subjects	Outcomes (Pre-Post Differences): Overbite		Overjet		SNA (°)		SNB (°)		ANB (°)		PP-SN (°)		Occl Pl (°)		MP-SN (°)		SN-U1 (°)		UL-E (mm)		LL-E (mm)		PP-U1 (°)		PP-U6 (°)		PP-U1 (mm)		PP-U6 (mm)		Stm-U1 (mm)		Duration		Root Resorption (mm)		Intrusion Rate (mm/month)		ANS-Me (mm)	
												M	SD	M	SD	M	SD	M	SD	M	SD	M	SD	M	SD	M	SD	M	SD	M	SD	M	SD	M	SD	M	SD	M	SD	M	SD	M	SD	M	SD	M	SD	M	SD
				18.75 ± 3.47(intrusive arch)					ar; Linear)	Connecticut intrusion arch	12	−2.04	1.37													0.08	0.51	2.50	5.11					1.92	5.23	2.25	2.99	−1.75	0.72	2.25	2.99	−2.00	1.15	5.8	2.9			0.3	0.18		
Gurlen, 2016	RCT	32	16 M; 16 F	14.65	NR	2, 1.4 X 7 mm	Between central and lateral	60gr for both	Cephalometric (Dental; Skeletal); Root Resorption	Mini-Screw	16	-3.27	0.86			-0.24	0.66	0.10	0.30	-0.34	0.74					0.31	0.98							9.38	3.51			-2.45	0.59												
										Connecticut intrusion arch	16	-2.05	1.09			-0.11	0.57	-0.20	0.53	0.09	0.66					0.80	1.46							6.62	3.36			-1.49	0.98												
Jain, 2014	RCT	30	11 M; 19 F	16-22	NR	2, 1.4 X 6 mm	Between central and lateral	1.5 ounces per side (80gr) for MS, UA; 2 for J hook	Overjet, Overbite, Cephalometric (Dental)	Mini-Screw	10	-2.33	1.00	-1.66	0.97																							-2.08	2.55	-0.17	2.91	-1.91	2.93	4	0						
										Head gear	10	-0.8	0.71	-0.8	2.22																							-0.1	2.21	0.2	1.45	-0.8	1.05	4	0						
										Utility Arch	10	-2	1.99	-0.42	1.85																							-1.33	2.71	0.75	2.18	-1.41	1.84	4	0						
Karagoz, 2013	RCT	25	11 M; 14 F	18.2 ± 3.3	NR	2, 1.4 X 8 mm	Between central and lateral	100 gr	Overjet, Overbite, Cephalometric (Dental; Skeletal); Root Resorption	Mini-Screw	13	-4.5	-8.2, -2.1	3.7	0.4, -5.5	-1.19	1.14	-0.28	1.2	-0.89	0.95					0.78	1.6	19.77	6.52									-4.18	1.65	0.13	1.16			4.5	4-7.5	-1.11	0.52				
										Segmental + TPA	12	-3.8	-4.5, -2.6	1.7	-0.9, -3.1	-0.47	1.04	0.08	1.08	-0.58	0.73					0.28	1.23	12.85	5.48									-3.04	0.92	0.38	0.93			5	4-7	-1.08	0.46				
Nayak, 2011	Prospective Cohort	14	NR	NR	NR	1, 2 X 8 mm	In the midline in the frenum region	50gr	Cephalometric (Dental)	Mini-Implant	7																	0.14	2.04									-3.29	1.11	0.29	0.49			6	0						
										Utility Arch	7																	7.86	9.14									-1.29	0.76	0.71	0.76			6	0						
Kumar, 2015	Prospective Cohort	30	NR	15-20	Class II Div 1	2, 1.3 X 7 mm	Between central and lateral	60gr for both	Cephalometric (Dental)	Mini-Screw	15																	0.93	1.27									-3.1	0.67	0.97	0.4			6	0						
										Connectic ut intru	15																	-3.73	1.28									-2.07	0.53	1.2	0.32			6	0						

Author Year	Study Design	Sample Size	Gender	Age	Malocclusion	Number of mini-screws	Mini-screw Placement	Force	Assessment	Group	Subjects	Outcomes (Pre-Post Differences)																																									
												Overbite		Overjet		SNA (°)		SNB (°)		ANB (°)		PP-SN (°)		Occl Pl (°)		MP-SN (°)		SN-U1 (°)		UL-E (mm)		LL-E (mm)		PP-U1 (°)		PP-U6 (°)		PP-U1 (mm)		PP-U6 (mm)		Stm-U1 (mm)		Duration		Root Resorption (mm)		Intrusion Rate (mm/month)		ANS-Me (mm)	
												M	SD	M	SD	M	SD	M	SD	M	SD	M	SD	M	SD	M	SD	M	SD	M	SD	M	SD	M	SD	M	SD	M	SD	M	SD	M	SD	M	SD	M	SD	M	SD	M	SD
										sion arch																																									
Liou, 2010	Retro Cohort	50	4M; 46F	25.4±5.6	NR	2, 2 X 9 mm	Infrazygomatic	100 gr intrusive, 250 gr retractive	Root Resorption	Mini-Screw Anchorage	30																																			16.0% to 20.0% (2.5-2.8 mm)					
										Control	20																																			13.4% to 14.4% (2.1-2.3 mm)					
Ma, 2013	RCT	20	NR	22.41 ±2.02	NR	2, 1.6 X 8 mm	Between central and lateral	50gr	Overjet; Overbite; Cephalometric (Dental; Skeletal)	Mini-Screw	10	-4.1	0.88	-0.11	0.05									-3.63	0.59	-2.33	0.92	1.57	0.28									-3.42	1.17	0.37	0.56	-3.21	0.87	5.28	0.82						
										Utility Arch	10	-3.76	0.78	0.10	0.07									-1.49	0.83	1.51	0.92	2.58	1.24									-2.95	0.78	0.51	0.17	-2.95	0.45	5.87	093						
Polat-Ozsoy, 2009	Prospective Cohort	24	10M; 14F	20.90 ±7.12 (mini-screw) 15.25 ±3.93 (utility arche)	NR	2, 1.2 X 6 mm	Between lateral and canine	50gr miniscrew	Overjet; Overbite; Cephalometric (Dental; Skeletal)	Mini-screw	13	-2.17	0.6	-0.32	2.31	-0.61	3.31	-0.11	2.46	-0.42	4.00					-0.19	1.10			0.12	2.78	-1.03	3.50	3.85	6.68			-2.98	3.06	0.15	0.65			6.61	2.95			0.44			
										Utility Arch	11	-2.32	0.4	0.82	2.79	-0.77	3.34	0.28	4.17	-1.05	1.67					-0.05	1.27			0.28	2.42	0.34	3.27	13.55	13.48			-1.81	2.87	1.8	0.78			6.61	2.46			0.27			
Senisik, 2012	RCT	45	19 M; 26 F)	20.29 ± 3.12	Class II Division 2	2, 1.3 X 5 mm	Between lateral and canine	90gr mini-screw; 60 gr CTA	Overjet; Overbite; Cephalometric (Dental; Skeletal)	Mini-Screw	15	-2.27	0.59	0.6	0.66									-2.77	1.64	-0.03	0.55							8.1	5.17	0	0	-2.47	0.81	0.3	0.13	-.09	1.39	6.93	1.17			0.34		-0.04	0.37
										Connecticut Intrusion Arch	15	-2.1	1.2	0.37	1.23									-2.73	1.5	0.3	07							4.87	5.64	9.83	3.53	-2.2	0.9	0.67	0.36	-0.77	1.21	6.88	0.95			0.31		0.12	0.32
										Control	15	0	0	0	0									0.13	0.48	-0.03	0.48							0	0	0	0	-0.17	0.94	0	0	0.13	0.81	6.9	1.01					0.03	0.4
Vela-Hernández, 2020	Retro Cohort	44	20 M; 24 F	36.6 ± 4.9	Class I	1/2, both 1.6 X 8 mm	In midline / Between lateral and canine	90gr per mini-screw	Overbite; Cephalometric (Dental; Skeletal)	One Mini-screw	16	-2.75	1.63													-0.3	1.95							14.3	9.99			-5.69	2.66							2.2	0.88				
										Two Mini-Screws	28	-3.8	1.43													0.61	2.15							11.58	8.03			-8.19	3.66							2.11	0.82				

Author Year	Study Design	Sample Size	Gender	Age	Malocclusion	Number of mini-screws	Mini-screw Placement	Force	Assessment	Group	Subjects	Outcomes (Pre-Post Differences)																																							
												Overbite		Overjet		SNA (°)		SNB (°)		ANB (°)		PP-SN (°)		Occl Pl (°)		MP-SN (°)		SN-U1 (°)		UL-E (mm)		LL-E (mm)		PP-U1 (°)		PP-U6 (°)		PP-U1 (mm)		PP-U6 (mm)		Stm-U1 (mm)		Duration		Root Resorption (mm)		Intrusion Rate (mm/month)		ANS-Me (mm)	
												M	SD	M	SD	M	SD	M	SD	M	SD	M	SD	M	SD	M	SD	M	SD	M	SD	M	SD	M	SD	M	SD	M	SD	M	SD	M	SD	M	SD	M	SD	M	SD	M	SD
Verma, 2020	Prospective Cohort	12	NR	29.5± 2.1	Class I or Class II Div 1 malocclusion	2, 1.3 X 8 mm	Between central and lateral	30gr each side	Cephalometric (Dental)	Mini-screw	6																									24.3	8.4	2.3	0.53	0.43	0.08									0.38			
										K-SIR loop	6																									17.5	7.5	1.9	0.57	0.53	0.02									0.31			
